# The Portosystemic Shunt for the Control of Variceal Bleeding in Cirrhotic Patients: Past and Present

**DOI:** 10.1155/2022/1382556

**Published:** 2022-09-17

**Authors:** Petre Radu, Virgiliu-Mihail Prunoiu, Victor Strâmbu, Dragos Garofil, Roxana Elena Doncu, Eugen Brătucu, Laurentiu Simion, Maria-Manuela Răvaş, Mircea Nicolae Brătucu

**Affiliations:** ^1^“Carol Davila” Hospital Surgery, Bucharest 010731, Romania; ^2^“Carol Davila” University of Medicine and Pharmacy, Bucharest 020021, Romania; ^3^Clinic I of General and Oncological Surgery, Bucharest, Romania; ^4^“Prof. Dr. Alexandru Trestioreanu” Oncological Institute, Bucharest 022328, Romania; ^5^Chair of Modern Languages, Bucharest 020021, Romania

## Abstract

Based on an experience of more than 50 years in the treatment of portal hypertension (PHT), the authors review and analyze the evolution of the surgical portocaval shunt (PCS). We would like to provide an insight into the past of PCS, in order to compare it with the current state of the treatment of PHT complications. As a landmark of the past, we shall present statistics of more than 500 cases of PHT operated between 1968 and 1983. From this group, 238 patients underwent surgical portocaval shunting during a fifteen-year period. The behavior of the portal hemodynamics following PCS was studied and the postoperative decrease in portal pressure (PP), as well as the residual PP, were recorded. The portal manometric determinations were made by electronic recordings using the Hellige device and direct intraoperative recordings through the catheterization of a ramus in the portal area. The results of PCS are superposable, in terms of hemodynamic efficiency, with those of the intrahepatic shunt (TIPS—transjugular intrahepatic portosystemic shunt). The authors discuss the current place of PCS, in obvious decline in comparison with the situation 50 years ago. The current methods of controlling variceal bleeding represent obvious progress. PCS remains with very limited indications, in specific situations when the other therapeutic methods have failed or are not recommended.

## 1. Introduction

The portal hypertension syndrome (PHT) is most of the time a component of the underlying pathology, i.e., hepatic cirrhosis. It is the hemodynamic component of cirrhosis. Thus, in hepatic cirrhosis, besides the alteration of portal hemodynamics, the following coexist liver failure, hepatic cytolysis syndrome, hepatic inflammation syndrome, metabolic syndrome, renal syndrome, etc. When PHT becomes clinically manifest through the occurrence of complications, it means that the hemodynamic impairment through hypervolemia and hypertension is dominant in the intra- and extrahepatic portal area. In most cases, the cirrhotic patient dies due to two complications liver failure and/or gastrointestinal bleeding (GIB).

The first cause of death, liver failure, results from the severe deterioration of the hepatic function, which is irreversibly altered. The second cause of death, GIB, is the consequence of the rupture of the esophageal and gastric varices. PHT continues to remain a permanent topic of interest in the medical world. This interest is due to one of the most dramatic complications accompanying PHT—variceal bleeding. Sudden bleeding in a cirrhotic patient remains one of the most challenging situations for the on-call team.

Different specialists participate and cooperate in the diagnosis and treatment of PHT: gastroenterologists, hepatologists, radiologists, and, increasingly seldom, surgeons. The severe occurrences of PHT are dominated by GIB, ascites, and portal-systemic encephalopathy (PSE). This is a neuropsychiatric disorder that occurs secondary to chronic liver disease. PSE is the result of ammonia intoxication of entero-portal origin. The cirrhotic liver is unable to metabolize the ammonium ion, which thus reaches the brain and determines its toxicity. It is manifested by confusion, loss of motor coordination, extreme agitation, and flapping tremor. All these manifestations are significant hepatic coma. To these, splenomegaly with hematological and immunological hypersplenism is added, in the initial stages of the disease. The volume and quality of the portal blood reaching the liver depend on the functional moments of the organs in the splanchnic area [1]. In this splanchnic area, the origin of the portal system, there are arterioportal anastomoses, sphincter-like structures, and adjustable resistances ensuring the regulation of the blood flow passing through the portal area on its way to the liver. The portal flow displays certain independence against the pressure and flows in the systemic circulation. Thus, the portal flow to the liver remains constant. A relatively constant blood volume reaches the sinusoidal capillary bed. In the liver, there are several sphincter-like structures arranged pre-post sinusoidally [2]. The interplay between these sphincter-like structures, including the arteriolar ones, acts like a “peripheral heart” on the “sinusoidal delta,” regulating the flow of the transhepatic blood.

To sum up, we can conclude that in PHT resulting from hepatic cirrhosis the following intrahepatic hemodynamic disorders occur: faulty suprahepatic drainage, the compression of the portal ramifications, the reduction of the sinusoidal capillary bed, the hyperplasia of the Kupffer cell mass, and the destruction of the sphincter system. All these make a mechanical obstacle to the transhepatic flow [3–6]. However, there is also a dynamic component represented by the arterial hyper flow: the increase of the hepatic and splenic flow, hepatic and gastric arterioportal anastomoses, and the destruction of the hepatic arteriolar sphincters. The portal system can be defined as a system with an adjustable capacity, being located between two capillary beds the splanchnic and the hepatic sinusoidal ones. Until 1988, the year when the transjugular intrahepatic portosystemic shunt (TIPS) was introduced by Rössle et al. [7, 8], the complications of PHT were mostly treated in the department of gastroenterology and surgery. The portocaval shunts (PCS) were the center of attention due to the fact that they enabled the performance of a remarkable portal decompression, which prevented the patient from developing GIB and ascites. In time, besides shunt surgery, a series of pharmacodynamic therapeutic methods and methods of endoscopic hemostasis of esophageal varices were developed and practiced. Beginning with the 1970s the first studies regarding the late results of PCS were published. The initial enthusiasm for wide portocaval derivations diminished starting with the 7th and 8th decades of the 20^th^ century. The immediate postoperative, as well as the late mortality, proved that the risks connected with liver failure and severe portal-systemic encephalopathy (PSE) were quite high following this type of surgery. Some called this type of surgery “the surgery of despair.” Direct troncular PCS with the greatest capacity for portal decongestion was blamed and so the procedure almost disappeared from the therapeutic resources in the 1980s. It was replaced with radicular shunts using the rami of the portal system of the splenorenal type (with its variants), as well as mesocaval shunts. They achieved a lower decompression of the portal area, but were not accompanied by liver failure through severe portal hypoperfusion or severe PSE.

## 2. Material and Method

The authors' intention is to present a point of view on the current place of PCS. We had the opportunity to access statistics drawn up 50 years ago, which included an ample study of portal manometry, conducted by the Department of Surgery of the Caritas Hospital of Bucharest, Romania, during the period 1968–1983. Thus, we had the possibility to make a foray into the past in order to provide a comparative view of the evolution of PCS over a 50-year period, from the surgical shunt until today's TIPS. So far this study has not been the object of any publication in the medical literature. All the determinations of portal pressure (PP) were performed by electronic manometry, using the Hellige recording system (Freiburg, Germany). The study was carried out under the supervision of D. Burlui and one of the authors of this paper [9, 10]. The portal manometry recordings were made intraoperatively through the direct catheterization of a portal ramus the repermeabilized ileal, splenic, or umbilical vein. The group included 550 patients with PHT who had undergone surgery in the Department of Surgery of the Caritas Hospital of Bucharest. The portal pressure (PP), normally directly measured in the portal system is 5–12 mmHg, about 4 mm higher than the free pressure in the hepatic veins. No measurements of the portohepatic gradient were carried out because the direct pressure recording in the portal area was preferred. Out of these 550 patients, 40% were hospitalized for GIB (220 patients), and 55% (302 patients) were hospitalized for ascitic decompensation. The remaining 28 patients (5%) were hospitalized for splenomegaly with hypersplenism.

Regarding the cause of PHT, 85,6% (470 patients) had an intrahepatic obstruction of the portal flow (cirrhosis), 7,8% (43 cases) had a prehepatic obstruction (thrombosis or portal cavernoma), 6,4% (35 cases) recorded an association of the two types of obstacles (cirrhosis and portal thrombosis), while 0,2% (1 case) presented with segmental thrombosis on the splenic vein. We would like to mention that the cirrhoses had postviral causes with hepatitis B virus (HBV) and hepatitis C virus (HCV) infections in 85% of the cases, while 15% were of nutritional origin. Regarding the staging of the cirrhotic patients, these were classified in accordance with the Child-Pugh score as follows: 23% class A, 32% class B, and 45% class C. At that time, the MELD (Model For End-Stage Liver Disease) score had not been introduced. If we refer to the complications of PHT, then the classification of the patients is made based on a variable scale with the degree of PHT [9–12]—Table 1.

## 3. Results

The mean value of the PP recorded directly in a ramus of the portal system in our patients was 25 mmHg. From the assessment of the values of the pressure, we found that they ascended from stage I to stage III A, and afterward they descended until stage III (Table 2). It is obvious that the onset of ascites, which defines stage III, marks a partial decrease of hypervolemia and portal stasis which is reflected in the PP. In the group we studied 50 years ago, GIB was present in 40% of the cases. This percentage included all the patients in stage II and some of those in stage III, who, besides ascitic decompensation, also presented with hemorrhage (Child B and C). The inferior limit of PP in the group with GIB was 22 mmHg. We did not register any GIB below this pressure value (Table 2). We also found that in stage III of PHT 50% of the patients in stage III A and only 16% of the patients in stage III B presented with bleeding. This decrease in PP in stage III B is clinically expressed through a decrease in the rate of GIB in patients with ascitic decompensation. Ascitic decompensation in stage III A and the progression of ascites in stage III B evolve inversely proportional to PP and GIB. Thus, it seems that the lymphatic decompression of the liver plays a certain part in the decrease of PP and of its hemorrhagic complications. The behavior of PP was also assessed according to the portal obstruction. Cirrhotic patients had a mean PP of 21.6 mmHg, while those with prehepatic obstruction had a mean value of 28.7 mmHg. There is a difference of 7.1 mmHg between the two types of portal obstruction (Table 2). The prehepatic obstructions generate a PP which is definitely higher than those present in the intrahepatic obstruction. This determines the higher severity of GIB in patients with prehepatic obstruction.

**Figure 1:**
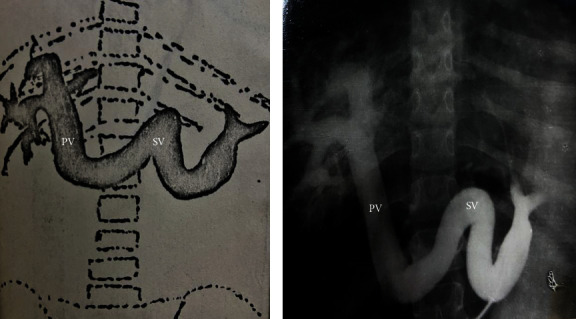
(a) and (b). Preoperative splenoportography (SPG) with the splenic vein (SV) angiomegaly in portal hypertension (PHT) with permeable portal trunk (PV—portal vein) and hepatogram of hepatic cirrhosis (*E. Brătucu*—Private collection).

**Figure 2:**
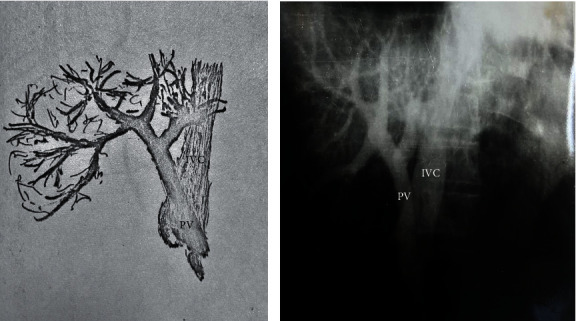
(a) and (b) PHT (portal hypertension) stage III A Ileoportography (IPG) after latero-lateral portocaval anastomosis. Functional anastomosis. Opacification of the inferior vena cava (IVC). Uninjected left hepatic lobe. Hepatoportal flow is present together with a discreet retro-hepatic narrowing of the vena cava. PV—portal vein. (*E. Brătucu*—*Private collection)*.

We had the opportunity to analyze the values of PP after a PCS, by following the behavior of the pressure according to the type of the shunt: the drop in PP and the postshunt residual PP. In the Department of Surgery of the Caritas Hospital, a number of 238 PCS procedures were performed in 15 years, as follows: termino-lateral troncular portocaval anastomoses (T-L TPCA)—83 patients, latero-lateral PCA (L-L PCA)—76 cases, splenorenal anastomoses (SRA)—65 cases, and mesocaval anastomoses—14 cases (Figures 1(a) and 1(b) and 2(a) and 2(b)). Apart from these, among the cases in our department, there were 312 splenectomies for splenomegaly with hematological hypersplenism, which were isolated splenectomies or associated with PCS. Measurements of the PP were performed across the whole group of 550 patients, including splenectomies. Before shunting, in the patients with PCS, a mean PP of 25.1 mmHg was recorded, while postshunting a mean PP of 13.5 mmHg was recorded. Thus, there was a drop in PP of 11.6 mmHg (Table 3). A simple splenectomy decompressed the portal system by only 2.2 mmHg. The level of the residual pressure after PCS—13.5 mmHg—situates the patient within a safe area, free from hemorrhagic and ascitic complications, the pressure level being only 2.5–3 mmHg higher than the normal one. It is interesting to analyze the mean PP according to the type of PCS. In patients with truncal PCA, we noted an average decrease of 10.6 mmHg; in those with T-L PCA, the average decrease was 13.6 mmHg, while in those with L-L PCA the decrease was 11.4 mmHg. In splenorenal and mesocaval PCS, the decrease in pressure was 7 mmHg (Table 3). By analyzing the postshunting residual pressure, regardless of the type of the shunt, we noted that this pressure of 13.5 mmHg ensured the prophylaxis of GIB or of the hemorrhagic relapse: the PP resulting after T-L PCA was 11.7 mmHg, after L-L PCA it was 15.8 mmHg, while after radicular anastomoses (splenorenal and mesocaval) it was 13 mmHg. It should be noted that PCS, regardless of the type, provides protection from GIB and ascitic decompensation (Table 3).

The post-PCS perioperative mortality (45 days) we recorded was 18.2%. No PCS were performed during a full hemorrhagic emergency. In such situations of acute bleeding, in the 1980s, transgastric ligatures of the varices associated with devascularization of the interazygoportal disconnection type were performed. The high mortality rate was due to postshunting liver failure, hepatorenal syndrome, and severe coagulation disorder. The best results were obtained with radicular derivations with 10% deaths and troncular PCS with 25% deaths. Shunting had similar mortality both for T-L PCA and L-L PCA. The lower rate of deaths after the proximal SRA of the Linton type, with tactic splenectomy—or after mesocaval derivation with H-graft—is partially explained by the performance of radicular derivations in stage II PHT, thus with better hepatic reserves than those of the patients in stage III PHT. The 5-year survival post-PCS was 31.4% in our group. In the literature of the time, the 5-year survival rate was 50–60% [11, 12, 19, 20]. For a casuistics of 400 PCS, Orloff [21, 22] gave 15-year survival rates of 57%, with a 5-year survival rate of 78%, portal-systemic encephalopathy (PSE) being 18%. A special note belongs to a group of surgeons led by M.S. Orloff, who published the result of a randomized study in 2001, examining comparatively 78 patients who had undergone a TIPS procedure, with a group of 76 patients with PCS, the procedures being performed in hemorrhagic emergencies.

The authors concluded definitely in favor of PCS regarding hemorrhagic relapse, the thrombosis of the anastomosis, the survival rates after 5 and 10 years, as well as postoperative encephalopathy. According to these authors, while TIPS enabled the relapse of GIB in 80% of the patients after one year, and the survival rate was 21% after 10 years, in the PCS group of patients, the relapse of GIB was below 1% after one year, while the survival rate was 60% after10 years. [22].

These results cannot be found in other authors. PSE after PCS developed in 43.2% of the patients we had operated on, yet it was corrected through protein restriction. PSE was present predominantly in the patients with PCS in the Child-Pough B or C stage. According to the stages of the evolution of PHT, we recorded the following percentages of postshunting PSE: 56% stage III, 36% stage II, and 8% stage I. Analyzing the frequency of PSE according to the type of PCS, we noted that it was present in 42% of the patients with T-L PCA and in 46% of those with L-L PCA. The increased incidence of PSE following L-L PCA concurs with the fact that this type of derivation was more frequently used in stage III of PHT, for the treatment of ascites. L-L PCA determines a hepatofugal decompression of the intrahepatic portal area, facilitating liver failure and encephalopathic impairment. The values of ammonemia as an indicator of PSE after PCS varied between 77 and 120 *μ*mol/l, with an average of 85 *μ*mol/l. The radicular shunts were not accompanied by PSE and ammonemia was within normal limits.

## 4. Discussions

Our past manometric records are still valid today. The methods of diagnosing and treating PHT have undergone a radical change. The emergence of noninvasive imaging techniques has led to visible progress in the approach to PHT. A first step was taken following the introduction of the treatment with beta-blockers for the prophylaxis and treatment of variceal GIB. Under pharmacodynamic therapy, the result is splanchnic vasoconstriction with a drop in the portal flow. There are also medicines that have the effect of reducing resistance to the transhepatic portal flow through intrahepatic vasodilatation.

The presence of esophageal varices has certainly determined direct interventions on them—endoscopic ligation. However, even under these circumstances of conservative treatment—beta-blockers and variceal banding—ever since the 1980s, open surgery has remained a last resort approach in cases when GIB could not be controlled or where it recurred after nonsurgical techniques [23–28]. The year 1988 marked a giant leap in the treatment of PHT—the introduction of the transjugular intrahepatic portal shunt—TIPS—into surgical practice, a procedure which has rapidly gained ground, becoming currently a standardized method of choice for the treatment of the complications of PHT. The merit belongs to the surgeons in Freiburg who promoted the method (Richter and Rössle) [7, 8]. At present, more than 30 years after the first TIPS, the indications and contraindications of the approach are well-established [29–37]. TIPS is accepted today as a solution prior to hepatic transplant, a “bridge” toward transplant. In fact, it is a method of invasive microsurgery. It proves its value when the control of hemostasis fails through conservative approaches. The creation of an alternative for vascular access in order to obtain a hemodynamic decompression of the hepatic portal and sinusoidal area is in fact a surgical act of altering vascular anatomy and of partial bypass of the sinusoidal bed with the aim of reducing the resistance to the transhepatic blood flow. In short, a portohepatic venous fistula is created. Extensive studies have sought to establish the place of TIPS and of surgical shunts in the management of GIB due to variceal rupture [29–36]. Often, the previously mentioned statistics offer contradictory data. Ample studies have detailed the advantages of PCS. Others are favorable to TIPS [37–40]. The disadvantages of TIPS are the high mortality after 45 days following the procedure, the modest 2-year survival rate, the recurrence of the bleeding, the obstruction of the stent, and the higher costs than PCS (Table 3).

Attempting to synthesize all the data, we propose the following points of view:

- TIPS is a valuable method of healing by first and second intention in variceal GIB, after the failure of pharmacotherapy and endoscopic hemostasis. All the studies clearly demonstrate the remarkable effect of TIPS in controlling GIB—85% hemostasis [36].
- TIPS significantly lowers the incidence of bleeding relapse, but it has a series of contraindications, according to AASLD (The American Association for the Study of Liver Disease—guidelines) [37] heart failure, uncontrolled systemic infections, severe pulmonary hypertension, obstructive jaundice, hepatic tumors, portal thrombosis, severe coagulopathy, thrombocytopenia below 20,000/m^3^ [37].
- TIPS is accompanied by the risk of developing PSE, of up to 31% higher than the risk following radicular shunts (splenorenal and mesocaval) [37].
- TIPS is a highly valuable technique and has priority in situations of the greatest emergency when conservative therapy cannot be used or has failed. In such circumstances, TIPS becomes a life-saving alternative [36, 37].

It is obvious that TIPS also has a maximum recommendation selectively, when there is no emergency, as a secondary prophylactic method for patients with Grade 2 esophageal varices [34, 38–40]. Then what is the current place of PCS in the treatment of variceal GIB? In emergency situations for GIB recurrence, if the conservative hemostasis and TIPS cannot be performed, we can resort to hemostasis through devascularization of the hepatogastric pole (interazygoportal disconnection, associated or not with the transgastric ligation of the cardiac varices). We used such therapeutic approaches in severe hemorrhagic emergencies in the 1980s. It is a quick and simple procedure, with an immediately visible hemostatic effect. Unfortunately, at present, it is only rarely found in the therapeutic arsenal of variceal bleeding [13–18].

The devascularizaton by azygoportal disconnection can be performed laparoscopically as well, as the interception of the vascular pedicles, the left gastric pedicle, and the short gastric vessels with the left gastroepiploic one being easy to achieve.

It is only very rarely that PCS preserves its indication and solely in patients with no hemorrhagic emergency, only when TIPS cannot be performed or the patients cannot qualify for TIPS (TIPS is obstructed or there is GIB due to prehepatic portal obstruction). At the same time, from the wide range of PCS procedures, only the radicular portosystemic derivations between the portal rami and the inferior cava area should be preserved: the splenorenal or mesocaval shunts. These can provide satisfaction even today, if they are performed as elective surgery, without any hemorrhagic emergency [41–49]. The examination of the data in our study reveals the long way from the open surgery of portosystemic derivations 50 years ago to the minimally invasive techniques of performing TIPS today. Likewise, the association of conservative therapy through beta-blocking medication and endoscopic variceal ligation has considerably changed the therapeutic approach to variceal bleeding. Thus, the surgical indications for the classical shunt have been drastically limited. The two methods of performing a shunt are beneficial interventions for the portohepatic hemodynamics impaired by the obstruction of the transhepatic flow. The decompression of the esophageal and gastric varices is obtained by facilitating the hepatic portal venous drainage. From our data, it can be noted that by using TIPS a similar result to that achieved by the surgeons in the 8^th^ decade of the 20^th^ century [50–53] can be obtained.

Shunts, regardless of the manner of performing them, either as TIPS or surgical PCS, produce their effects as a result of the decrease of PP at a level below the value of hemorrhagic risk. There are also surgical techniques that do not aim at lowering PP, but resort to the decompression of the esophageal varices by performing a disconnection between the portal and azygos areas, the azygoportal disconnection (APD). This therapeutic procedure has a long and quite considerable history behind it. The first APDs were performed by Tanner [54], and subsequently, in 1966, by Torres and Degni [55]. In a nutshell, the APD techniques achieve an interception of the communication between the portal vascular areas, which supply and maintain the esophageal varices, and the system of the azygos vein. “AtSugiura and Hassab types” grammatically unclear. Please rephrase the sentence for clarity and correctness. “ present, this type of procedure is performed in surgeries of the Sugiura and Hassab [53] type. APDs are indicated in hemorrhagic emergencies for patients who did not respond well to conservative treatment, if TIPS is contraindicated, it has failed or it is unavailable. The indications for ADP are splenomegalies with hypersplenism, unavailable or inefficient TIPS, and extensive portal thrombosis. ADP presupposes splenectomy and an extensive devascularization of the upper gastric pole, and, subsequently, it can be associated with the banding of esophageal varices, if necessary. ADPs have a series of advantages that have led to their promotion by surgeons, i.e., there are techniques that can also be performed laparoscopically, with low mortality and morbidity rates, and without episodes of encephalopathy. Quite frequently ADPs are performed by surgeons in the Asiatic region, who appreciate their therapeutic performances [53, 56–58].

Recently, a comparative study between the laparoscopic ADP and the classic PCS of the splenorenal or mesocaval type has been published. The efficiency of stopping the bleeding is similar—3.6% bleeding recurrences. They recorded a longer time for the ADP surgeries, a similar postsurgical PSE (0.8%), as well as a similar perioperative death rate—2.4%. Other authors from Asiatic countries have also communicated encouraging data for ADP: a 97% survival rate after 5 years, bleeding recurrence of 2.4%, and postoperative mortality of 4% [59–62].

## 5. Conclusions

To conclude, the classic shunt surgery remains with strictly limited recommendations for use in patients who do not have an acute hemorrhagic episode, with hepatic reserves (Child A and Child B), and who do not qualify for TIPS or liver transplant. We have attempted to offer an objective picture of the evolution of the portocaval shunt during these 50 years, from a method with broad indications to a technique with only limited application. This obvious rebound of PCS was due to the advancement of less invasive methods, TIPS, and azygoportal disconnection.

## Figures and Tables

**Table 1:** Stages of PHT.

Stage	Classification	Percentage
Stage I	Splenomegaly with hypersplenism	12% (66 patients)
Stage II A	Esophageal varices	33% (182 patients)
Stage II B	Variceal bleeding	
Stage III A	Pharmacodynamically reversible ascites	55% (302 patients)
Stage III B	Pharmacodynamically irreversible ascites	

**Table 2:** PP variation according to the stage of PHT.

Stage I	15.2 (mmHg)
Stage II A	21.6
Stage II B	24.1
Stage III A	25.3
Stage III B	22.0

STD—stage; PP- portal pressure; PHT—portal hypertension.

**Table 3:** Comparison PCS—TIPS.

PCS Caritas Hospital—experimental study		TIPS	
PRE—shunt PP	25.1 mmHg	>12 mmHg	
Remaining PP	13.5 mmHg	<12 mmHg	
Pressure drop	11.6 mmHg	?	
Postshunt EP	43.2%-APC 0%-ARS	20–30%	[13–18]
Recurrent GIB	1%	27%	
Postoperatively. Mortality (45 days)	18.2% 10%-ASR	23–29%	
1-year mortality	1%	17–20%	
Ineffective shunt	0%	3–7%	
5 years survival	31.4%	60%	

PCS—portocaval shunt; TIPS—transjugular intrahepatic portosystemic shunt; PSE—portal-systemic encephalopathy; PP—portal pressure; GIB—gastrointestinal bleeding; SRA- splenorenal anastomosis; TPCA—troncular portocaval anastomosis (direct).

## Data Availability

All the data given in the article are correct. Unfortunately, 50 years ago, there were no computers to do statistics, and part of the primary data is lost, leaving the conclusions that were communicated in this article, together with the authors' experience over time.
